# A novel syndrome of lethal familial hyperekplexia associated with brain malformation

**DOI:** 10.1186/1471-2377-12-125

**Published:** 2012-10-27

**Authors:** Mohammed Zein Seidahmed, Mustafa A Salih, Omer B Abdulbasit, Meeralebbae Shaheed, Khalid Al Hussein, Abeer M Miqdad, Abdullah K Al Rasheed, Anas M Alazami, Ibrahim A Alorainy, Fowzan S Alkuraya

**Affiliations:** 1 Neonatology Unit, Department of Pediatrics, Security Forces Hospital, Riyadh, Saudi Arabia; 2 Division of Pediatric Neurology, Department of Pediatrics (39), College of Medicine, King Saud University, P. O. Box 2925, Riyadh, 11461, Saudi Arabia; 3 Division of Pediatric Neurology, Department of Pediatrics Security Forces Hospital, Riyadh, Saudi Arabia; 4 Developmental Genetics Unit, Department of Genetics, King Faisal Specialist Hospital and Research left, Riyadh, Saudi Arabia; 5 Department of Radiology and Diagnostic Imaging, King Khalid University Hospital and College of Medicine, King Saud University, Riyadh, Saudi Arabia; 6 Division of Genetics, Department of Pediatrics, King Khalid University Hospital and College of Medicine, King Saud University, Riyadh, Saudi Arabia; 7 Department of Anatomy and Cell Biology, College of Medicine, Al Faisal University, Riyadh, Saudi Arabia

**Keywords:** Hyperekplexia, Microcephaly, Simplified gyral pattern, Cerebellar underdevelopment, Autosomal recessive

## Abstract

**Background:**

Hyperekplexia (HPX) is a rare non-epileptic disorder manifesting immediately after birth with exaggerated persistent startle reaction to unexpected auditory, somatosensory and visual stimuli, and non-habituating generalized flexor spasm in response to tapping of the nasal bridge (glabellar tap) which forms its clinical hallmark. The course of the disease is usually benign with spontaneous amelioration with age. The disorder results from aberrant glycinergic neurotransmission, and several mutations were reported in the genes encoding glycine receptor (GlyR) α1 and β subunits, glycine transporter GlyT2 as well as two other proteins involved in glycinergic neurotransmission gephyrin and collybistin.

**Methods:**

The phenotype of six newborns, belonging to Saudi Arabian kindred with close consanguineous marriages, who presented with hyperekplexia associated with severe brain malformation, is described. DNA samples were available from two patients, and homozygosity scan to determine overlap with known hyperkplexia genes was performed.

**Results:**

The kindred consisted of two brothers married to their cousin sisters, each with three affected children who presented antenatally with excessive fetal movements. Postnatally, they were found to have microcephaly, severe hyperekplexia and gross brain malformation characterized by severe simplified gyral pattern and cerebellar underdevelopment. The EEG was normal and they responded to clonazepam. All of the six patients died within six weeks. Laboratory investigations, including metabolic screen, were unremarkable. None of the known hyperkplexia genes were present within the overlapping regions of homozygosity between the two patients for whom DNA samples were available.

**Conclusions:**

We present these cases as a novel syndrome of lethal familial autosomal recessive hyperekplexia associated with microcephaly and severe brain malformation.

## Background

Hyperekplexia (HPX) was first described in 1958 by Kirstein and Silverskoild [1] who reported a family with emotionally precipitated drop seizures. It is a rare non-epileptic disorder characterized by generalized stiffness immediately after birth which normalizes during the first two to three years of age [2], exaggerated persistent startle reaction to unexpected auditory, somatosensory and visual stimuli, and non-habituating generalized flexor spasm in response to tapping of the nasal bridge (glabellar tap) which is the clinical hallmark of HPX [3,4]. Minimum stimuli may provoke severe jerky movements of all limbs, but the course of the disease is usually benign with spontaneous amelioration with increasing age [2]. The exaggerated startle response however, persists to adulthood leading to unprotected falls without loss of consciousness [5].

Genetic etiology of HPX has long been suspected because many cases were familial and although autosomal dominant inheritance was the most commonly observed mode of inheritance, sporadic cases as well as kindreds consistent with autosomal recessive inheritance have also been reported [6,7]. Several loci have been identified by linkage analysis on 5q33 – q35 [8], 4q31.3, 14q 24 [9], 11p15.2-15.1 [10], and Xq11.1 [11] but it was the identification of the actual disease genes in these loci that provided the much needed molecular insight into the pathology of HPX. We now know that HPX is a disorder of glycinergic neurotransmission after several mutations were reported in the genes encoding glycine receptor (GlyR) α1 and β subunits, glycine transporter GlyT2 as well as two other proteins involved in glycinergic neurotransmission gephyrin and collybistin [12]. *GlyT2* mutations identified by Rees et al. [10], which are associated with severe neonatal apnea episodes, included homozygous or compound heterozygous mutations, as well as a heterozygous mutation (in one patient) consistent with autosomal dominant inheritance. On the other hand, it should be noted that mutations in *ARHGEF9*, encoding collybistin and *GPHN*, encoding gephyrin, are not commonly associated with hyperekplexia. Rather, mutations in *ARHGEF9* were reported to cause X-linked intellectual disability [13-16] whilst mutations in GPNH are associated with molybdenum co-factor deficiency [17,18].

Glycine receptors are ligand-gated chloride channels that cause postsynaptic hyperpolarisation and synaptic inhibition in the brainstem and spinal cord. They are clustered, together with specific subtypes of GABA_A_ receptors, at neuronal postsynaptic membranes by the action of gephyrin, a protein that is translocated to the cell membrane by the GDP–GTP exchange factor collybistin [9,11]. Mutations that either prevent the surface expression of, or modify the function of, the heteromeric α1 and β subunits of the glycine receptor (GlyR) chloride channel manifest as HPX [19].

In this report we describe a novel syndrome of lethal familial autosomal recessive HPX affecting six newborns, and associated with gross brain malformation characterized by severe simplified gyral pattern and cerebellar underdevelopment.

## Methods

Six patients from two branches of a large consanguineous Saudi Arabian family were enrolled in the study. An informed written consent was used to recruit the patients and their relatives (King Faisal Specialist Hospital and Research left [KFSHRC] IRB-approved research protocol [RAC #2080006]. The study adhered to the tenets of the Declaration of Helsinki. Written consent to publish the images and videos was obtained.

Genomic DNA was extracted from whole blood and processed for genotyping on the Axiom Chip platform as per the manufacturer’s protocol (Affymetrix, Santa Clara, CA, USA). Resulting genotypes were processed for runs of homozygosity using autoSNPa (dna.leeds.ac.uk/autosnpa) and homozygosity scan to determine overlap with known hyperkplexia genes was performed as described [20].

## Results

**Figure 1:**
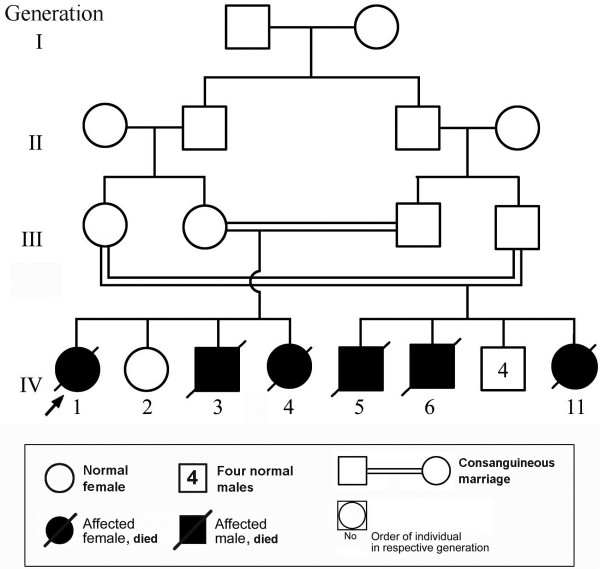
**Pedigree of the family under study with the box (below) containing the key to the pedigree symbols.** The arrow indicates the proband.

The studied large consanguineous family (Figure 1) consisted of 2 sibships and the disease manifested in the 4^th^ generation where six children were affected. Demographic, clinical and salient investigations of these are provided in Table 1.

**Table 1:** Phenotypic features of the patients

**Patient No (Pedigree No, Figure 1)**	**Patient 1** **( IV.1)**	**Family 1**	**Patient 3 (IV.4)**	**Patient 4 (IV.5)**	**Family 2**	**Patient 6 (IV.11)**
		**Patient 2 (IV.3)**			**Patient 5 (IV.6)**	
Sex	F	M	F	M	M	F
Gestational age	Term	Term	Term	Term	Term	Term
Excessive fetal movement	+	+	+	NA	+	+
Birth weight (gm)	2985	3200	2620	NA	2980	2970
Occipitofrontal circumference (cm)	30.5	32	31	NA	31	31
**Signs of hyperekplexia**						
• Stiffness	+	+	+	+	+	+
• Non-habituating generalized flexor spasm to glabellar tap	+	+	+	+	+	+
• Exaggerated persistant Startle reaction to stimuli	+	+	+	+	+	+
**Major neurologic findings**						
• Microcephaly	+	+	+	+	+	+
• Hypertonia	+	+	+	NA	+	+
• Arthrogryposis	+	+	+	NA	+	+
Ophthalmic examination	Bilateral optic atrophy	Bilateral optic atrophy	Bilateral optic atrophy	NA	Bilateral optic atrophy	Bilateral optic atrophy
Electroencephalography (EEG)	• Background: Widespread low voltage brain waves, with occasional synchronized jerks mostly myogenic in origin.	• Background: Consisted of low voltage brain wave tracing all over the record.	NA	NA	• Background: Diffuse slow activity with low voltage for age.	EEG: Full of motion artifacts but the background was continuous without epileptic discharges.
		• Prominent electrical contamination from the ventilator.			• No spikes or sharp waves and no abnormal focal changes.	
		• Occasional synchronized jerks most likely coming from the muscle.			• No electrical sign of seizure activity.	
Metabolic screen	Negative	Negative	Negative	NA	Negative	Negative
Chromosome analysis	46XX	46XY	46XX	NA	46XY	46XX
CT brain	Figure 2A & B: Brain underdevelopment with large extra cerebral CSF spaces	Figure 2C: Remarkable brain under-development with very few sulci in frontal and temporal lobes. Sylvian fissures are under-developed and wide open.	Figure 2E & F: Remarkable cerebral and cerebellar under-development, simplified gyral pattern and enlargement of extra cerebral CSF spaces.	NA	Cerebral and cerebellar atrophy	NA
MRI brain	NA	Figure 2 D: Small cerebellar vermis.	NA	NA	NA	Figure 2 G & H: Simplified gyral pattern. The frontal and temporal lobes are very few and shallow. Sylvian fissures are wide open.
Age at death	6 weeks	2 weeks	6 weeks	4 days	4 weeks	3 weeks

+ = Present, NA = Not available.

### Clinical report

#### Patient 1

The proband (Figure 1, IV.1), a Saudi girl, was born by uncomplicated vaginal delivery at term. Pregnancy was marked with excessive fetal movements with no associated poly- or -oligodramnios. Antenatal screen was normal. The mother, 20-year-old primigravida, was healthy and had no significant medical problem. Parents were first cousins, the father was 23-years-old and healthy. The proband was followed by a normal 5-year- old female sibling (Figure 1, IV.2) and two similarly affected children: Patient 2 (Figure 1, IV.3) and Patient 3 (Figure 1, IV.4).

She was ventilated shortly after birth for apnea following abnormal jerky movements and stiffness, and was started on intravenous (IV) phenobarbitone.

Examination: Birth weight 2985 gm (50^th^ centile), length 47 cm (10^th^ centile), head circumference 30.5 cm (below 5^th^ centile). No dysmorphic features apart from microcephaly with small anterior fontanelle. Neurologic examination showed increased tone, spasticity and exaggerated startle reflex. She was very sensitive to sounds and minimum stimuli provoked severe jerky movements of all limbs. There was also sustained non-habituating glabellar tap 'Additional movie files show this in more detail [see Additional files 1 and 2]'. She had arthrogryposis of both upper and lower limbs. Ophthalmic examination revealed bilateral optic atrophy.

Laboratory investigations showed normal electrolytes, renal and hepatic functions and the hematologic indices were normal. Creatinine kinase (CK) was normal. Tandem mass spectrometry (TMS) for metabolic disorders screen was unremarkable. Serum ammonia, lactate and urine organic acids were normal. Chromosome analysis showed normal female karyotype 46XX. The background of EEG trace consisted of widespread low voltage brain waves, with occasional synchronized jerks, mostly myogenic in origin.

On account of frequent tonic/clonic movements without alteration of consciousness, she was commenced on clonazepam which controlled the movements, but she developed frequent apneic episodes; and was intubated and connected to ventilator support. She remained ventilator dependent and developed severe sepsis to which she succumbed at the age of 6 weeks.

**Figure 2:**
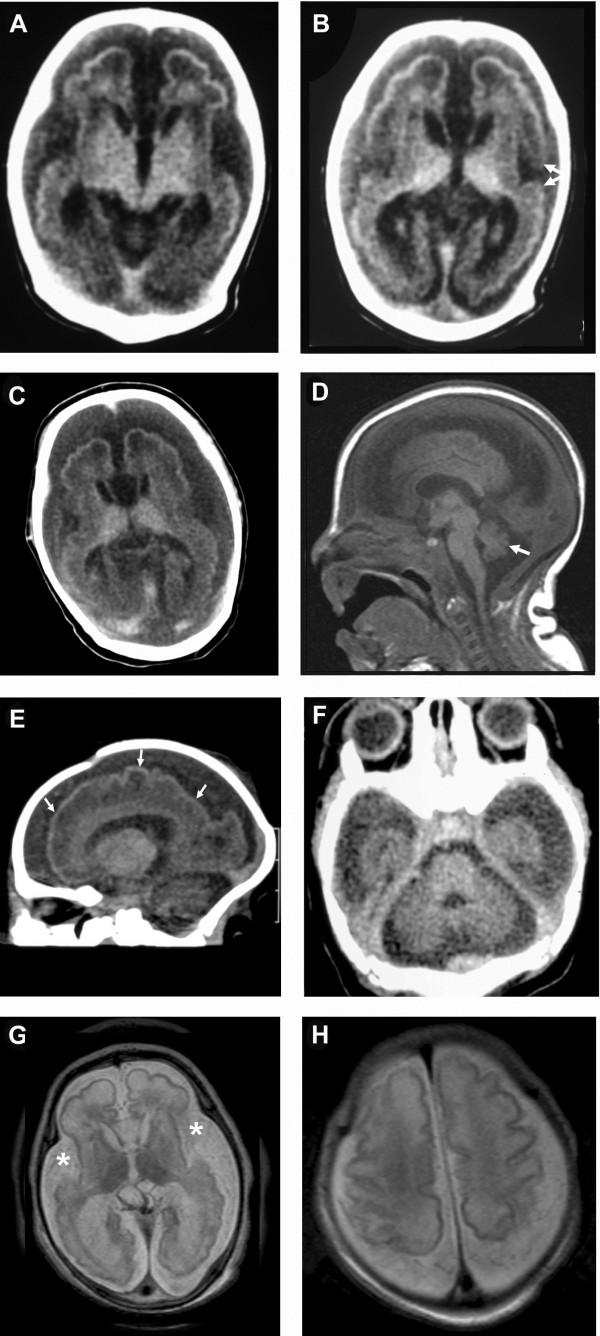
**Patient 1 (Figure**1**, IV.1) CT axial images of the brain (A&B, age** = **21 days) demonstrating brain underdevelopment with large extracerebral CSF spaces.** The frontal and temporal lobes sulci [**A**] are more developed compared to Patient 6 [G&H], but still immature. The Sylvian fissures [**B**] are more developed and have visible frontal and temporal opercula (arrows). [C&D]. Patient 2 (Figure 1, IV.3). [**C**] CT image of the brain (age = 3 days) showing remarkable brain underdevelopment, very few sulci in the frontal and temporal lobes, and large extracerebral CSF spaces. The Sylvian fissures are underdeveloped and wide open. [**D**] Sagittal T1-weighted MR image of the brain demonstrating small cerebellar vermis (arrow) and enlarged cisterna magna and CSF spaces in the posterior fossa. The corpus callosum is fully developed and the brain stem has normal appearance. [E&F]. Reconstructed sagittal [**E**] and direct axial [**F**] CT images of the brain (Patient 3: Figure 1, IV.4) at the age of four days demonstrating remarkable cerebral and cerebellar underdevelopment evident by simplified gyral pattern (arrows) and enlargement of extracerebral CSF spaces. [G&H] Axial T2-weighted MR images of the brain (Patient 6: Figure 1, IV. 11) at the age of one day showing simplified gyral pattern. The sulci of the frontal and temporal lobes are very few and shallow [**G**], while the Sylvian fissures are wide open (asterisk). Better gyration and deeper sulci are seen in the brain convexity around the Rolandic fissure **[H]**. The extracerebral CSF spaces are remarkably enlarged.

Brain CT scan, done at the age of 21 days (Figure 2A and B), showed marked brain atrophy with mildly dilated ventricles, abnormal configuration and lissencephaly with smooth surface and microgyria. The frontal and temporal lobes sulci were still immature.

#### Patient 2

A male newborn (Figure 1, IV.3), brother of Patient 1, was delivered normally at term. His mother had gestational diabetes (GDM) during pregnancy which was treated with insulin. There were excessive fetal movements. Apgar scores were 7 and 8 at one and five minutes, respectively. Birth weight 3200 g and head circumference 32 cm (below 5^th^ centile). Examination revealed the same phenotype as his affected sibling (Patient 1) which consisted of microcephaly, exaggerated startle reflex in response to non-habituating glabellar tap and violent jerky movements provoked by sounds and touch (see Additional file 3).

Laboratory investigations, including metabolic screen, were unremarkable and chromosome analysis showed normal male karyotype. The EEG trace showed low voltage background, occasional synchronized jerks (myogenic), and no epileptiform discharges.

He had repeated episodes of stiffness and apnea which were managed by clonazepam and mechanical ventilation. However, he developed severe sepsis and more tonic/clonic jerks, for which he was started on IV phenobarbitone with poor response. He died at the age of two weeks.

CT image of the brain (Figure 2C), done at the age of three days, revealed remarkable brain underdevelopment with very few sulci in the frontal and temporal lobes, and large extracerebral cerebrospinal fluid (CSF) spaces. The Sylvian fissures were underdeveloped and wide open. Brain MRI (Figure 2D) showed small cerebellar vermis, enlarged cisterna magna and CSF spaces in the posterior fossa, fully developed corpus callosum, and normal appearing brain stem.

#### Patient 3

This baby girl (Figure 1, IV.4) was delivered normally at term. Pregnancy was marked by excessive fetal movements and antenatal ultrasound revealed microcephaly. Birth weight was 2620 gm (25^th^ centile) length 44 cm (5^th^ centile), head circumference 31 cm (5^th^ centile). Apgar score was 7 and 9 at one and five minutes, respectively. Examination revealed no dysmorphic features apart from microcephaly and arthrogryposis of both upper and lower limbs and stary anxious look. Neurologic examination revealed increased tone, stiffness and non-habituating sustained glabellar tap which provoked violent rhythmic jerky movements with flexion of both upper and lower extremities and exaggerated startle reflex to tactile and auditory stimuli. Ophthalmic examination revealed bilateral optic atrophy and no neurocutaneous abnormalities were detected.

Laboratory investigations were unremarkable and included metabolic screen by TMS and urine for organic acids, and s.sulphocystine (for isolated sulphite oxidase and molybdenum cofactor deficiency). Chromosome analysis revealed normal female karyotype. TORCH screen for congenital infections was negative. Electroencephalography (EEG) was not done.

The baby was managed by clonazepan with good response regarding the stiffness and the frequency of abnormal movements but she remained ventilatory dependent and died at the age of 6 weeks.

Brain CT (Figure 2E and F), at the age of four days, demonstrated remarkable cerebral and cerebellar underdevelopment evident by simplified gyral pattern and enlargement of extracerebral CSF spaces.

#### Patient 4

This baby boy (Figure 1, IV. 5) was delivered at another hospital. Parents are first cousins and brother and sister of parents of Patients 1–3 (Figure 1). The parents reported that the baby had small head and abnormal jerky movements and died at the age of 4 days. No clinical, laboratory or radiological data were available.

#### Patient 5

Brother of Patient 4 (Figure 1, IV. 6) was delivered normally at term, and pregnancy was unremarkable apart from excessive fetal movements. Birth weight 2980 g (25^th^ centile), head circumference 31 cm (below the 3^rd^ centile), and Apgar scores were 6 and 7 at one and five minutes, respectively. He manifested with symptoms of hyperekplexia and responded to external stimuli with jittery movements that became rhythmic and mimicked seizures.

Examination, showed no dysmorphic features apart from microcephaly and arthrogryposis of all limbs. He also showed spasticity, exaggerated startle reflex with severe tonic clonic movements in response to glabellar tap which was non-habituating. Ophthalmic examination showed bilateral optic atrophy with retinal dysplasia and abnormal macula.

Laboratory investigations that were unremarkable included metabolic screen, TORCH panel for congenital infection, and chromosome analysis which showed normal male karyotype. The background of the EEG showed diffuse slow activity with low voltage. There were no spikes or sharp waves and no abnormal focal discharges.

He developed repeated episodes of apnea necessitating intubation and assisted ventilation, was managed by clonazepam and phenobarbitone, but died at the age of 4 weeks. Brain CT revealed severe cerebral and cerebellar underdevelopment (image not shown).

#### Patient 6

A female sister (Figure 1, IV. 11) of Patients 4 and 5 (Table 1) was delivered normally at term. Pregnancy was marked by excessive fetal movements. Antenatal ultrasound scan revealed microcephaly and there were abnormal fetal movements during the scan. Apgar scores were 5 and 8 at one and five minutes, respectively. Birth weight 2970 g (50^th^ centile), length 47 cm (10^th^ centile), and head circumference 31 cm (below 5^th^ centile).

Examination showed microcephaly, sloping forehead with low hair line, and arthrogyposis of both upper and lower limbs. Neurologic examination revealed increased tone, stiffness of the limbs and body, exaggerated startle reflex in response to slightest handling, non-habituating glabellar tap provoking excessive rhythmic jerky movements followed by apnea, bradycardia and desaturation. Ophthalmic examination showed bilateral optic atrophy.

Laboratory investigations were unremarkable and included metabolic screen, TORCH panel and chromosome analysis. EEG showed motion (myogenic) artifacts but the background was continuous without epileptic discharges.

She was managed by clonazepam to which she responded in the way of less frequent abnormal movements and stiffness. Because of frequent episodes of apnea and bradycardia, she was intubated and supported by mechanical ventilation. She died at the age of 3 weeks.

Brain MRI (Figure 2 G and H), done at the age of one day, revealed simplified gyral pattern. The sulci of the frontal and temporal lobes were very few and shallow, while the Sylvian fissures were wide open. Better gyration and deeper sulci were seen in the brain convexity around the Rolandic fissure, whereas the extracerebral CSF spaces were remarkably enlarged.

### Homozygosity analysis

None of the known hyperkplexia genes were present within the overlapping regions of homozygosity between the two patients for whom DNA samples were available (data not shown). Work is underway to identify the novel disease gene using a combination of homozygosity scan and exome sequencing as described before [21].

## Discussion

Neurologic symptoms and signs in the present cohort revealed the diagnostic criteria of hyperekplexia [2-4] namely, abnormal excessive fetal movements, stiffness immediately after delivery, exaggerated startle reflex and non-habituating exaggerated head retraction reflex (HRR) resulting in violent rhythmic jerks and breath holding episodes. They also had congenital hip dislocation. In addition, they had microcephaly and documented optic atrophy in all of the five ascertained patients. The EEG, done in four of six patients, showed no epileptic discharges, and the background was characterized by diffuse slow activity with low valtage for age, and synchronized jerks mostly myogenic in origin.

The brain imaging in early life in five of these patients (Figure 2) showed a constant finding of brain underdevelopment and immaturity manifested by simplified gyral pattern and enlargement of the CSF spaces. The frontal and temporal lobes had very few gyri and shallow sulci giving the lobes a smooth appearance [22]. This was less remarkable in the frontoparietal region close to the Rolandic fissure. The Sylvian fissures were open wide and not operculized in two patients. The cortical thickness was normal. The occipital horns of the lateral ventricles were enlarged giving the appearance of colpocephaly. The cerebellum in these patients was generally small with poorly developed folia and large cisterna magna. The brain stem was normal as well with no signs of underdevelopment or hypoplasia.

Our cases fulfilled all clinical criteria to be diagnosed as HPX [2,4,23-31]. They also showed good response to clonazepam, the drug of choice being a GABA agonist. Nevertheless, they differed from all the reported cases of HPX in that they were associated with microcephaly, optic atrophy and severe brain malformations. Also unlike the usually favorable prognosis of HPX in humans, the present cases had universally lethal outcome, and all the six patients died within 6 weeks.

The pedigree of our patients is consistent with autosomal recessive inheritance, although the number of affected individuals is vividly higher than the ratio predicted by classical mendelian genetics. Nevertheless, this is most likely an unfavorable outcome of a purely probabilistic 25% recurrence risk. We have observed this frequently in autosomal recessive neurogenetic disorders in this Region with high rate of consanguinity [32]. None of the known hyperkplexia genes were present within the overlapping regions of homozygosity between the two patients for whom DNA samples were available. Homozygosity scan and exome sequencing, as described before [21], are underway to identify the disease causing mutation in this family.

Hereditary hyperekplexia (HPX) is characterized by features consisting of generalized stiffness immediately after birth, normalizing during the first years of life. The stiffness increases with handling and disappears during sleep [23]. There is also fetal posture with clenched fists and anxious stare [2]. Excessive startle reflex to unexpected auditory, somatosensory and visual stimuli, is present at birth. Consciousness is unaltered during startle response. The tonic spasms mimic generalized tonic seizures leading to apnea and may lead to death [24]. Non-habituating exaggerated head-retraction reflex (HRR) elicited by tapping the tip of the nose, forehead (glabellar tap) or face [4,25,26], is considered by some as a clinical hallmark for diagnosis. Other associated features include periodic limb movements in sleep and hypnogogic myoclonus [5,27], and inguinal, umbilical or epigastric hernias due to increased intra-abdominal pressure [28]. Congenital dislocation of the hip, sudden infant death syndrome (SIDS) [29] and abnormal intrauterine fetal movements [30] have also been reported.

Diagnosis of HPX is mainly clinical, and all laboratory tests are typically normal including CT and MRI of the brain. Electroencephalogram (EEG) is usually normal but may show fast spikes (myogenic origin) initially during the tonic spasm followed by slowing of the background activity with eventual flattening corresponding to the phase of apnea, bradycardia and cyanosis. Electromyography (EMG) shows a characteristic almost permanent muscular activity with periods of electrical quietness, whereas nerve conduction velocity is normal [28].

Unlike the universally lethal outcome observed in our cases, the prognosis of HPX in humans is usually favourable with spontaneous amelioration of the hypertonia with increasing age but delayed gross motor development is usually observed. The tone is usually almost normal by the age of 3 years although hypertonia may recur in adult life. The exaggerated startle response, however, persists to adulthood leading to unprotected falls without loss of consciousness. Shahar et al. [31] reviewed the outcome of 39 cases of sporadic HPX during the neonatal period and early infancy and reported that the debilitating symptoms of HPX gradually resolved in all 39 infants treated with low dose of clonazepam. However, and in contrast to human hyperekplexia/startle disease, *GlyT2* mutations result in early neonatal lethality in mice, cows and dogs [33,34].

Malformations of cortical development, a prominent feature of the present cohort, constitute a diverse group of structural brain disorders reflecting deranged neuronal proliferation, migration or organization [35]. Recently, mutations in one gene (*WDR62*) were reported to cause a wide spectrum of severe cerebral cortical malformations including microcephaly, simplified gyral pattern, callosal abnormalities, and cerebellar hypoplasia [36-38]. Nevertheless, the reported phenotype of *WDR62* gene mutations did not include HPX. On the other hand, Goraya et al. [39] reported a case of HPX in a girl with posterior fossa malformation. Another case with unusual presentation of cerebral dysgenesis in a neonate associated with hyperekplexia was reported by Parveen et al. [40] but the clinical course and EEG findings favoured startle epilepsy secondary to cerebral dysgenesis as the diagnosis in the neonate they reported.

## Conclusions

We report six Saudi Arabian patients, product of consanguineous marriages, who presented in fetal life with excessive abnormal movements and in the immediate postnatal period with microcephaly, hyperekplexia responsive to clonazepam, associated with severe brain underdevelopment, characterized by simplified gyral pattern and small cerebellum, and optic atrophy of autosomal recessive inheritance with lethal outcome. To the best of our knowledge, this entity has not, hitherto, been described.

## Competing interests

The authors declare that they have no competing interests.

## Authors’ contributions

MZS and MAS conceived of the study, and participated in its design and coordination and drafted the manuscript. OBA, MS, KAH, AMM, AKR and IAO participated in management of patients and data analysis. AMA carried out the molecular genetic studies. FSA designed and coordinated the molecular genetic studies and critically reviewed the manuscript. All authors read and approved the final manuscript.

## Pre-publication history

The pre-publication history for this paper can be accessed here:

http://www.biomedcentral.com/1471-2377/12/125/prepub

## Supplementary Material

Additional file 1 **MOV (QuickTime) showing non-habituating glabellar tap in Patient 1 (Figure **1**, IV.1).** Click here for file

Additional file 2 **MOV (QuickTime) shows exaggerated startle reflex to sounds in Patient 1 (Figure **1**, IV.1).** Click here for file

Additional file 3 **MOV (QuickTime) showing abnormal tactile response with jittery movements that became rhythmic and mimicked seizures in Patient 2 (Figure **1**, IV.3).** Click here for file
